# [Expression of Concern] Delphinidin induces cell cycle arrest and apoptosis in HER-2 positive breast cancer cell lines by regulating the NF-κB and MAPK signaling pathways

**DOI:** 10.3892/ol.2026.15565

**Published:** 2026-04-01

**Authors:** Ailin Wu, Yanfeng Zhu, Bin Han, Jiayuan Peng, Xiaomin Deng, Wei Chen, Jingchang Du, Yu Ou, Xiaoli Peng, Xiaoping Yu

Oncol Lett 22: 832, 2021; DOI: 10.3892/ol.2021.13093

Following the publication of the above paper, it has been drawn to the Editor's attention by a concerned reader that the western blots shown for Cyclin B1 in Fig. 2B were strikingly similar to the data shown for the c-Raf-1 blots in Fig. 4C; in addition, the blots shown for the PKCα and IKKβ experiments in Fig. 5A were also remarkably similar.

The authors have been contacted by the Editorial Office to offer an explanation for these apparent anomalies in the presentation of the data in this paper, and we are awaiting their response. Owing to the fact that the Editorial Office has been made aware of potential issues surrounding the scientific integrity of this paper, we are issuing an Expression of Concern to notify readers of these potential problems while the Editorial Office continues to investigate this matter further.

